# Coexisting left ventricular aneurysm and pseudoaneurysm after inferior wall myocardial infarction

**DOI:** 10.1093/ehjci/jeaa103

**Published:** 2020-05-19

**Authors:** Iman Karaji, Øyvind Bleie, Kaia Skromme, Cedric Davidsen

**Affiliations:** Department of Heart Disease, Haukeland University Hospital, PO Box 1400, N-5021 Bergen, Norway

A 74-year-old man was admitted with an acute deterioration after 14 days of dyspnoea and coughing. His electrocardiogram showed Q-waves and minor ST-elevation in inferior wall leads, suggestive of a myocardial infarction. He was transferred to our centre for coronary angiography, which displayed an occluded mid-right coronary artery and a proximal stenosis followed by a chronic total occlusion in the mid-left anterior descending artery. Transthoracic echocardiography revealed a large ventricular aneurysm in the basal inferior wall (*Panel A* and Supplementary data online, *Moving Image A*), with a small defect with bidirectional flow arising from the apical part of the aneurysm (*Panels B* and *C*, and Supplementary data online, *Moving Image B*).

Cardiac computed tomography exposed an inferolateral aneurysm measuring 57 × 32 × 44 mm (*Panels D* and *E*, and Supplementary data online, *Moving Image E*). Apically in the left ventricular aneurysm a channel led to an anteriorly located pseudoaneurysm inside the pericardium. Also present was pericardial effusion with high density, suggestive of bleeding. After discussion in the heart team, the patient underwent aneurysm resection and coronary bypass surgery. He was discharged the 11th postoperative day and is currently doing well.

**Figure:**
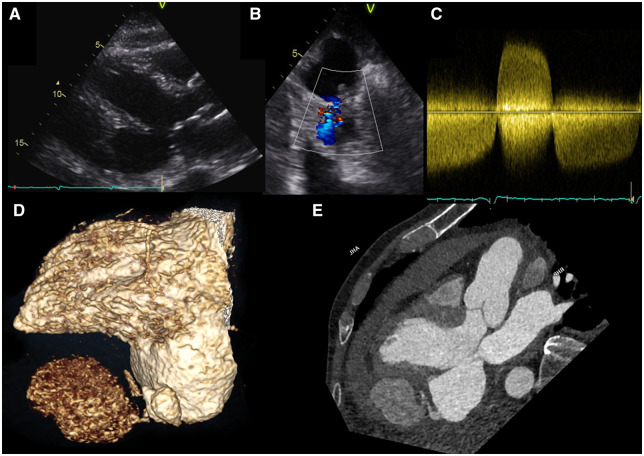


Left ventricular aneurysm, an evagination of all myocardial layers, may form as early as 5 days after a myocardial infarction. In comparison, a pseudoaneurysm is a contained rupture of the ventricle, which in this rare case itself originated from a left ventricular aneurysm. We suspect our patient’s aneurism developed after a silent transmural myocardial infarction 14 days prior to admission, with a subsequent contained rupture explaining his acute deterioration.

Supplementary data are available at *European Heart Journal - Cardiovascular Imaging* online.

## Supplementary Material

jeaa103_Supplementary_Data

